# Dietary diversity and possible cataract among Chinese elderly population

**DOI:** 10.3389/fnut.2024.1342190

**Published:** 2024-01-26

**Authors:** HaiYue Zhao, Junyang Zhang, Jie Zhou, Yinghui Ma

**Affiliations:** School of Economics and Management, Jiangsu University of Science and Technology, Zhenjiang, China

**Keywords:** dietary diversity, cataract, plant-based diet, older adults, China

## Abstract

**Background:**

While cataracts, the vision-clouding eye disease associated with aging, have long presumed dietary underpinnings, the relationship between dietary variety and cataract risk in developing nations has been nebulous. This research aims to investigate the association between dietary diversity scores (DDS) and the risk of cataracts, while considering various dietary diversity patterns.

**Methods:**

This research utilized cross-sectional data from 2008 to 2018 extracted from the Chinese Longitudinal Healthy Longevity Survey (CLHLS), implementing the Visual Function Index-14 (VF-14) to gauge cataract probability. The researchers captured participants' diet diversity by using the DDS metric and categorized it into total, animal-based, and plant-based diet patterns. To explore associations between dietary variety and cataract potential, a generalized estimating equation (GEE) was statistically modeled using the data, with adjustments made to account for potentially confounding factors. Additionally, sensitivity analyses were conducted, excluding individuals with assorted eye conditions, to isolate cataract relationships.

**Results:**

The study sample comprised 47,395 participants with a mean age of 86.1 years. The study found that a lower likelihood of developing cataract was correlated with both total diet (OR = 0.74; 95% CI: 0.69–0.79) and plant-based diet (OR = 0.65; 95% CI: 0.61–0.71), whereas a slightly higher risk was associated with animal-based diet (OR = 0.90; 95% CI = 0.84–0.96). The results remained unchanged in the sensitivity analysis.

**Conclusion:**

The diversified diets are linked to a decreased likelihood of developing cataracts, but animal-based diet faced heightened cataract odds. The implementation of a varied dietary regimen has the potential to serve as a cost-effective and efficient intervention strategy for the prevention of cataracts.

## 1 Introduction

Cataract is a prevalent ocular disease caused by the opacification of the transparent lens within the ocular structure, resulting in visual impairment and blindness among elderly individuals (1). According to the World Health Organization (WHO), cataract contributes to 47.9% of reported cases of worldwide blindness and visual impairment (2). While the occurrence of cataracts varies across regions and age groups, the majority of cases are recorded among individuals aged 60 years and older (reported prevalence: 1% in the under-40 age group and 88.17% in the over-60 age group) (3). Although cataracts may not be serious in the early stages, they will develop into blindness without early intervention. Research indicates that delayed treatment is the primary cause of visual impairment for patients with congenital or pediatric cataracts (4). Therefore, early diagnosis and treatment of cataracts are crucial to prevent further visual impairment and improve wellness of the older adults (4, 5).

Cataract development is influenced by a range of factors, including age, heredity, and external risk factors. Smoking, diabetes, and UVB exposure are consistently identified as risk factors (6, 7). Other potential risk factors include plasma constituents, steroids, alcohol, hypertension, hyperlipidemia, and obesity (8). Certain drugs, such as steroids, nifedipine, and heavy smoking, are associated with a higher risk, while aspirin-like analgesics and cyclopenthiazide may offer protection (9). However, the role of dietary factors in prevention remains unclear (8). Further research is needed to fully understand the complex interplay of these factors in cataract development (6).

Cataracts have substantial effects on various aspects of health and wellbeing, including but not limited to reading ability, driving proficiency, and self-care capabilities (10). Furthermore, empirical evidence also suggests that the presence of cataracts may potentially increase the likelihood of experiencing falls, fractures, depressive symptoms, and social isolation (11). Traditional diagnostic methods rely on fundus exams and visual acuity tests, diagnosing only once vision dips below 20/40 (12). However, the expert equipment and skills required make such evaluations impractical for widespread community screening. Additionally, subclinical cataracts that conventional methods fail to identify may have impact on visual function and quality of life (13). The Visual Function Index-14 (VF-14) as a perceived visual function measure holds great potential to address the issue of subclinical cataracts (14). The VF-14 is a simple, user-friendly, and compliant measure of perceived visual function, developed by the US National Institute of Ophthalmology. It is widely used to evaluate the QoL, cost-effectiveness, and outcomes of cataract patients' interventions (15). Cataract prevalence according to the VF-14 criteria varies from 0.42% to 2.05% in low-income countries and from 0.63% to 13.6% in high-income countries (16). This research uses part of the VF-14 questionnaire to identify elderly Chinese subclinical cataracts as “possible cataracts.” This study aims to promote early adjustment of harmful dietary habits, which may contribute to cataract prevention and wellness improvement.

Dietary factors may affect the development of cataract by influencing the oxidative stress in the lens. And it is widely believed that antioxidant nutrients are the key factors (17). For example, the connection between specific antioxidant nutrients (such as vitamin B1, vitamin A, lutein, etc.) and cataract risk has been proven (18). The results claim that higher intake of these antioxidant nutrients is associated with lower incidence and severity of cataract (19). Therefore, researchers now aim to unlock the protective powers of antioxidant-rich foods, believing these supercharged ingredients may safeguard the lens from oxidative damage (20). But previous research on the connection between dietary variables and the development of cataracts has been hampered by a few key issues (21–29). First, most research has concentrated on single nutrients or individual meals, neglecting potential interactions and synergy between dietary components (20–24). Second, owing to data restrictions, self-reported food consumption data are commonly employed. Thus, measurement mistakes and memory bias are widespread (24, 26). Third, most research has been done in wealthy nations, which may have different diets and cataract rates than developing nations (21–29).

To fully expose the antioxidant nutrient's protective effects on the lens from oxidative damage, it is necessary to investigate the overall diet or dietary diversity. The prospective Dietary Diversity Score (DDS) measures dietary diversification. The level of dietary diversity is determined by combining DDS scores for different foods. Higher DDS indicates a more varied and balanced diet (19, 28–31). DDS is reliable for assessing dietary nutritional adequacy and quality, according to researchers. DDS, as a simple tally of consumed food groups, has emerged as a powerful tool for unraveling connections between nutrition and illness. Researchers have utilized DDS to illuminate diet's impacts on a sprawling spectrum of conditions: obesity, diabetes, hypertension, cognitive decline, and even mortality (25, 32, 33). Studies show inverse relationships between DDS and chronic diseases: as diet diversity decreases, disease risk increases. Each added food group may help prevent conditions like CVD, cancer, and diabetes, where mechanisms remain uncertain. While DDS reveals nutrition variety's broad impact on health, further research on diet nuances could offer targeted nutritional remedies based on individual risk factors. DDS also improves health and longevity (29, 31). Previous studies have examined the association between adherence to the Dietary Guidelines for Americans (measured by HEI-2015 scores) and risks of gout and hyperuricemia using data from the National Health and Nutrition Examination Survey (NHANES). These studies have found that higher HEI-2015 scores are associated with decreased risks of these diseases. The HEI-2015 score considers multiple aspects of diet quality, including adequacy and moderation of food components. In this regard, it is similar to the dietary diversity score (DDS) which also focuses on diet diversity. Therefore, the application of HEI-2015 in previous studies demonstrates that using scoring methods that capture multiple dimensions of diet quality such as DDS to assess diet-health outcome relationships is reasonable. This helps to support the utility of diversity-focused dietary scores like DDS (34). In addition, empirical evidence also links DDS to improved cognitive performance, fewer physical limitations, and lower psychological stress in elderly adults (35, 36).

Despite cataracts' toll on global aging populations, few studies have probed associations between DDS and cataract risk, particularly in developing nations (19, 37, 38). This research gap proves troubling given the afflictions posed to China's massive elderly community. Though this demographic faces amplified cataract susceptibility, scarce data has explored the phenomenon among Chinese seniors (38–40). Therefore, this pioneering study seeks to illuminate the intricacies between cataracts and different kinds of diet diversities in China's vulnerable older populations. By unraveling relationships between nutrition variety, gender, and cataract prevalence, findings could unveil dietary remedies to empower China's elderly to proactively protect their vision and wellness. While research overall remains limited, this investigation aims to catalyze further scholarship on targeted nutritional interventions against cataracts across high-risk demographics worldwide.

## 2 Materials and methods

### 2.1 Study population

**Figure 1:**
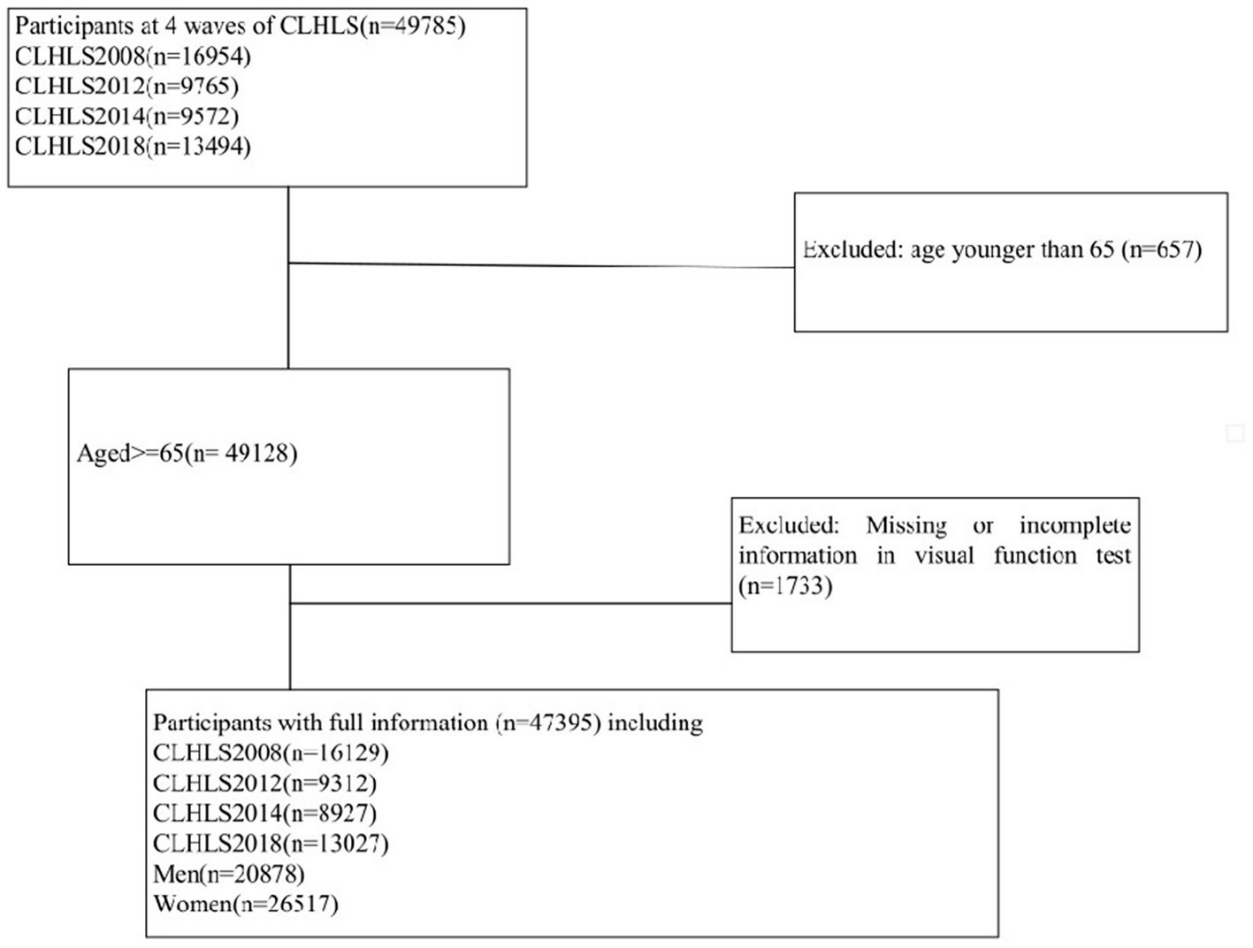
Flowchart of participants selection.

This study utilized the data from the Chinese Longitudinal Healthy Longevity Survey (CLHLS) to examine the relationship between dietary diversity and cataract risk in the elderly population in China. The CLHLS is a nationwide survey that covers the majority of regions in China. As shown in Figure 1, this expansive investigation drew from a robust sample of 49,785 participants surveyed over the decade spanning 2008 to 2018. In chronological order, 16,954 subjects completed assessments in the 2008 cohort, followed by 9,765 in 2012, 9,572 in 2014, and culminating with 13,494 seniors surveyed in 2018. Following comprehensive vision evaluations, we excluded 657 participants under 65 years old, as well as 1,733 individuals with incomplete or missing data. This exhaustive filtering process ensured an optimized final sample of the target elderly demographic, enabling more authoritative analyses of associations between nutrition, age, and cataract prevalence. Finally, with quality data from 47,395 seniors, we could shed light on dietary strategies to safeguard vision among China's most vulnerable populations. Informed consent was obtained from all participants and/or their families, and the study was approved by the Ethics Committee of Peking University (IRB00001052-13074).

### 2.2 Assessment of dietary diversity

To quantify nutritional diversity, we developed a 10-point DDS aligned with national Chinese dietary guidelines recommending intake from 10 food groups (29, 41, 42). Utilizing food frequency questionnaires covering cereals, vegetables, fruits, legumes, nuts, meat, eggs, fish, dairy, and fungi, we assigned 1 point for “regular” or “almost daily” consumption of each food category. This scoring system enabled total DDS calculations for each participant on a 0–10 scale. Beyond the total DDS, we also evaluated animal-based and plant-based DDS (43). Animal-based DDS tracked meat, fish, eggs, and dairy, with consumption frequency scored from 0–4. Plant-based DDS spanned six categories—grains, vegetables, fruits, legumes, nuts, and fungi—rated on 0–6 scales based on intake regularity. With these multi-faceted DDS techniques, we could illuminate the nuanced contributions of diverse food groups, from animal vs. plant sources to overall nutritional variety. These insights into the unique dietary diversity patterns could help inform targeted nutritional interventions to avoid age-related vision loss. To enhance the survey methodology, validated attention-recall questions were incorporated alongside traditional questionnaires. This addition aimed to stimulate precise recollections of dietary habits. For instance, participants were tasked with tasks such as reproducing a figure displayed on a card or recalling a set of three words provided to them earlier. From the research methods and results, there exists certain similarity between the HEI-2015 scores adopted by previous studies and the DDS scores. Specifically, HEI-2015 contains 9 adequacy components and 4 moderation components, which can evaluate the overall dietary quality and diversity. Similarly, DDS usually considers the number of food groups or categories to assess diet diversity. Previous studies have found that the high score groups of HEI-2015 and DDS are both associated with improvement in various health outcomes, such as reduced risk of sleep disorders, just like the high score groups of DDS, which are beneficial to cure chronic diseases like diabetes. Therefore, although previous studies did not explicitly examine DDS, their methods and conclusions provide evidence that HEI-2015 and DDS scores have certain comparability in reflecting dietary quality and the association with health outcomes (34, 44).

### 2.3 Assessment of possible cataract

To identify potential cataract cases, we deployed a self-reported visual function assessment adapted from the validated VF-14 questionnaire on cataracts' daily impacts (15). This simple yet insightful screening technique presented participants with a circle containing a break, then asked them to rate their ability to locate the break on a 4-point scale. Those rating their vision as a 1 (“can see and distinguish”) were classified as cataract-free, while scores of 2 (“can see only”) or 3 (“cannot see”) indicated potential cataracts. And we excluded individuals rating themselves as 4 (“blind”) (45). By relying on participants' perceptions of their own functional vision rather than clinical testing, this questionnaire-based approach enabled efficient, large-scale screening for cataract prevalence. The nuanced 4-point scale also illuminated gradations in impairment, differentiating total blindness from mild or severe cataract symptoms. With a validated technique tailored to elderly self-reporting, investigators could rapidly identify participants potentially afflicted by cataracts for further analysis of associations with nutrition.

### 2.4 Covariates

In order to facilitate a more authoritative examination of the link between different diets and cataract prevalence, researchers adjusted for a diverse spectrum of co-varying participant traits that could confound analyses of the isolated diet-cataract relationship. Analyzed participant traits ranged from gender (male or female), segmented age groups (65–79 years, 80–99 years, 100 years or older), education level (educated or uneducated), marital status (married or unmarried/divorced/widowed), pre-retirement occupation (peasant or non-peasant), and household income (<100,000 yuan or ≥100,000 yuan) to lifestyle factors like smoking status (never, previous or current), alcohol consumption patterns (never, previous or current), and physical activity levels (never, previous or current). Adjusting for this extensive set of co-varying factors enabled the isolation of the diet-cataract link for more authoritative analysis of how nutritional diversity alone relates to age-linked visual changes.

### 2.5 Statistical analysis

The data, presented as mean ± standard deviation or percentages, underwent chi-square testing to analyze categorical variable relationships across groups and independent *t*-tests to evaluate numerical variable differences. Utilizing Generalized Estimating Equation (GEE) models incorporating auto regressive (AR) working correlation structures, we calculated cataract development odds ratios (OR) across DDS quartiles. Model 1 was the unadjusted model. Building on the initial model, subsequent models integrated additional controls. Model 2 adjusted for demographic variables including gender, age, and household income to address confounding. Then, Model 3 incorporated further factors like education level, marital status, and pre-retirement occupation. Finally, Model 4 also accounted for lifestyle elements, including physical activity, smoking, and drinking patterns. A sensitivity analysis was performed to assess the reliability of the estimates after removing older individuals with eye conditions that affect vision. The data analysis was conducted using STATA statistical software version 17.0, which was specifically developed for the Windows operating system. The statistical significance was assessed using a two-tailed *p*-value of 0.05.

## 3 Results

### 3.1 Descriptive characteristics

The 47,395 participants exhibited 17,919 participants (37.8%) possible cataract cases, with a gender breakdown of 20,878 (44.1%) men and 26,517 (55.9%) women, as is shown in Table 1. Marital status included 17,579 (37.1%) married individuals. A vast array of 14,869 participants ranging from 65 to 79 years old are involved; an even more substantial cohort of 24,508 aged individuals between 80 and 99 provided their information; additionally, a particularly remarkable group of 8,018 extraordinarily aged folks 100 years of age or older offered their information. Education levels proved relatively low, with 45.7% of the participants uneducated. As for pre-retirement occupation, 28,853 people (60.9%) resided in urban areas. In addition, 46,280 participants (97.6% of the total), reported a household income <100,000 yuan. Regarding lifestyle factors, 16.6% were smokers, 15.9% drank alcohol, and 29.8% performed physical activities. As demonstrated in Table 1, the highest quartile (Q4) of the animal-based DDS contained not only the greatest absolute number but also the highest percentage of possible cataract cases at 2,608 and 14.6% respectively; in contrast, Q4 of the plant-based DDS Quartile contained the fewest number and lowest percentage of possible cataracts cases at 1,510 and 8.4% respectively, while total DDS Quartile Q4 displayed moderate numbers and percentages of possible cataracts at 2,151 and 12.0%.

**Table 1:** Baseline characteristics of all participants by cataract status.

**Variables**	**Total sample (*n* = 47,395)**	**Possible cataract (*n* = 17,919)**	**Non-cataract (*n* = 29,476)**	***P***
**Gender (%)**				<**0.001**
Male	20,878 (44.1)	6,224 (34.7)	14,654 (49.7)	
Female	26,517 (55.9)	11,695 (65.3)	14,822 (50.3)	
**Age (year) (%)**				<**0.001**
65–79	14,869 (31.4)	2,610 (14.6)	12,259 (41.6)	
80–99	24,508 (51.7)	9,943 (55.5)	14,565 (49.4)	
≥100	8,018 (16.9)	5,366 (29.9)	2,652 (9.0)	
**Education level (%)**				<**0.001**
Educated	25,741 (54.3)	12,222 (68.2)	13,519 (45.9)	
Uneducated	21,654 (45.7)	5,697 (31.8)	15,957 (54.1)	
**Marital status (%)**				<**0.001**
Married	17,579 (37.1)	4,088 (22.8)	13,491 (45.8)	
Unmarried, divorced, or widowed	29,816 (62.9)	13,831 (77.2)	15,985 (54.2)	
**Pre-retirement occupation (%)**				<**0.001**
Peasant	18,542 (39.1)	6,303 (35.2)	12,239 (41.5)	
Non-peasant	28,853 (60.9)	11,616 (64.8)	17,237 (58.5)	
**Household income (yuan) (%)**				<**0.001**
≥100,000	1,115 (2.4)	492 (2.7)	623 (2.1)	
<100,000	46,280 (97.6)	17,427 (97.3)	28,853 (97.9)	
**Smoking status (%)**				**0.008**
Previous	7,116 (15.1)	2,430 (13.7)	4,686 (16.0)	
Current	7,811 (16.6)	2,052 (11.6)	5,759 (19.7)	
Never	32,082 (68.2)	13,278 (74.8)	18,804 (64.3)	
**Consuming alcohol (%)**				**0.011**
Previous	5,899 (12.6)	2,158 (12.2)	3,741 (12.8)	
Current	7,460 (15.9)	2,038 (11.5)	5,422 (18.6)	
Never	33,511 (71.5)	13,510 (76.3)	20,001 (68.6)	
**Physical activities (%)**				**0.013**
Previous	4,709 (10.1)	2,153 (12.2)	2,556 (8.8)	
Current	13,937 (29.8)	3,367 (19.1)	10,570 (36.3)	
Never	28,133 (60.1)	12,131 (68.7)	16,002 (54.9)	
**Total DDS quartile (%)**				<**0.001**
Q1 (≤ 4)	17,413 (36.7)	7,382 (41.2)	10,031 (34.0)	
Q2 (>4 ≤ 5)	8,272 (17.5)	3,127 (17.5)	5,145 (17.5)	
Q3 (>5 ≤ 7)	14,433 (30.5)	5,212 (29.1)	9,221 (31.3)	
Q4 (>7 ≤ 10)	7,209 (15.2)	2,151 (12.0)	5,058 (17.2)	
**Animal-based DDS quartile (%)**				<**0.001**
Q1 (≤ 1)	14,237 (30.0)	5,827 (32.5)	8,410 (28.5)	
Q2 (>1 ≤ 2)	11,990 (25.3)	4,449 (24.8)	7,541 (25.6)	
Q3 (>2 ≤ 3)	13,782 (29.1)	5,035 (28.1)	8,747 (29.7)	
Q4 (>3 ≤ 4)	7,386 (15.6)	2,608 (14.6)	4,778 (16.2)	
**Plant-based DDS quartile (%)**				<**0.001**
Q1 (≤ 2)	15,564 (32.8)	6,762 (37.7)	8,802 (29.9)	
Q2 (>2 ≤ 3)	15,864 (33.5)	6,114 (34.1)	9,750 (33.1)	
Q3 (>3 ≤ 4)	10,080 (21.3)	3,445 (19.2)	6,635 (22.5)	
Q4 (>4 ≤ 6)	5,761 (12.2)	1,510 (8.4)	4,251 (14.4)	
*N*	47,395	17,919	29,476	47,395

DDS, dietary diversity score.

### 3.2 DDS and possible cataract

The odds of possible cataract were calculated using GEE models, considering both the overall DDS and the type of nutrition sources. The initial analysis showed that those in the highest quartile of DDS had a lower probability of having possible cataract compared to those in the lowest quartile. Specifically, total diet (OR = 0.59; 95% CI: 0.56–0.62), animal-based diet (OR = 0.80; 95% CI = 0.75–0.85), and plant-based diet (OR = 0.47; 95% CI = 0.44–0.51) showed significant associations with lower odds of potential cataract. In the subsequent model (Model 2 and Model 3), when we adjusted for more covariates including gender, age, household income, education level, marital status, and pre-retirement occupation, all associations and signs of coefficients remained unchanged. Final exhaustive adjustments for smoking, alcohol, and activity habits (Model 4) lead total diet (OR = 0.74; 95% CI: 0.69–0.79) and plant-based diet (OR = 0.65; 95% CI: 0.61–0.71) to retain their marked shielding effects. However, alarmingly, the animal-based diet relationship reversed to show heightened cataract odds (OR = 0.90; 95% CI: 0.84–0.96) in Table 2, marking a concerning departure from initial trends. Generally, ORs below 1 confirm animal-based diet associated with reduced cataract odds overall. But, from Q2 to Q4, animal-based diet ORs increased from 0.88 to 0.90, implying rising cataract risk as diversity grew. Moreover, animal-based diet ORs exceeded plant-based at all quartiles. For instance, Q4 animal-based diet showed higher cataract odds (OR = 0.90) than Q4 plant-based (OR = 0.65). These trends imply animal foods uniquely lack the pronounced cataract protection seen with plant-based diet. The results capture nuanced synergies, suggesting animal-based diets may increase cataract risk through inflammation or glycation despite general benefits of diet variety.

**Table 2:** Odds ratios of possible cataract by quartiles of DDS among whole samples.

**Variables**	**Quartiles of DDS**				**P trend**
	**Q1**	**Q2**	**Q3**	**Q4**	
Total DDS	(0, 4]	(4, 5]	(5, 7]	(7, 10]	
Model 1	1.00	0.83^***^ (0.79, 0.88)	0.78^***^ (0.75, 0.82)	0.59^***^ (0.56, 0.62)	<0.001
Model 2	1.00	0.84^***^ (0.79, 0.89)	0.81^***^ (0.77, 0.85)	0.64^***^ (0.60, 0.69)	<0.001
Model 3	1.00	0.85^***^ (0.80, 0.91)	0.84^***^ (0.79, 0.88)	0.68^***^ (0.64, 0.73)	<0.001
Model 4	1.00	0.87^***^ (0.82, 0.93)	0.87^***^ (0.82, 0.91)	0.74^***^ (0.69, 0.79)	<0.001
Animal-based DDS	(0, 1]	(1, 2]	(2, 3]	(3, 4]	
Model 1	1.00	0.87^***^ (0.82, 0.91)	0.85^***^ (0.81, 0.89)	0.80^***^ (0.75, 0.85)	<0.001
Model 2	1.00	0.84^***^ (0.79, 0.89)	0.85^***^ (0.80, 0.89)	0.79^***^ (0.74, 0.84)	<0.001
Model 3	1.00	0.86^***^ (0.81, 0.90)	0.88^***^ (0.83, 0.93)	0.85^***^ (0.79, 0.91)	<0.001
Model 4	1.00	0.88^***^ (0.83, 0.93)	0.91^***^ (0.86, 0.96)	0.90^**^ (0.84, 0.96)	<0.001
Plant-based DDS	(0, 2]	(2, 3]	(3, 4]	(4, 6]	
Model 1	1.00	0.83^***^ (0.79, 0.87)	0.69^***^ (0.65, 0.72)	0.47^***^ (0.44, 0.51)	<0.001
Model 2	1.00	0.89^***^ (0.84, 0.93)	0.74^***^ (0.70, 0.78)	0.57^***^ (0.53, 0.62)	<0.001
Model 3	1.00	0.90^***^ (0.85, 0.94)	0.76^***^ (0.72, 0.80)	0.60^***^ (0.56, 0.65)	<0.001
Model 4	1.00	0.92^**^ (0.88, 0.97)	0.79^***^ (0.75, 0.84)	0.65^***^ (0.61, 0.71)	<0.001

Model 1 included DDS as the sole variable; Model 2 further controlled for gender, age, and household income; Model 3 additionally controlled for education level, marital status, and pre-retirement occupation; Model 4 added difficulty in physical activities, smoking, and drinking; DDS, dietary diversity score.

^***^, ^**^, and ^*^ represent significance levels of 1%, 5%, and 10%, respectively.

### 3.3 Subgroup analyses

Delving into gender-stratified analyses through Model 4 in Table 3 reveals intriguing insights: total diet traced strong shielding effects both among men (OR = 0.74; 95% CI: 0.67–0.83) and women (OR = 0.74; 95% CI: 0.68–0.81) to the risk of getting cataracts. Plant-based diets followed suit, with matched robust benefits for men (OR = 0.68; 95% CI: 0.60–0.77) and women (OR = 0.64, 95% CI: 0.58–0.71). However, a concerning divergence emerged for animal-based diets. While women saw no significant links, men faced heightened cataract odds (OR = 0.86; 95% CI: 0.78–0.96), marking an alarming departure from initial trends. This gender-specific reversal introduces nuance, suggesting animal-based diets uniquely threaten men despite general protective effects across both genders for total and plant-based diets. Similar patterns of divergence between animal-based and plant-based diets were also evident in the results across various age groups.

**Table 3:** Association between quartiles of DDS and possible cataract.

**Variables**	**Quartiles of DDS**				**P trend**
	**Q1**	**Q2**	**Q3**	**Q4**	
**Men**					
Total DDS	1.00	0.86^**^ (0.79, 0.95)	0.88^**^ (0.81, 0.96)	0.74^***^ (0.67, 0.83)	<0.001
Animal-based DDS	1.00	0.81^***^ (0.74, 0.88)	0.87^**^ (0.80, 0.95)	0.86^**^ (0.78, 0.96)	<0.001
Plant-based DDS	1.00	0.93 (0.86, 1.01)	0.84^***^ (0.77, 0.93)	0.68^***^ (0.60, 0.77)	<0.001
**Women**					
Total DDS	1.00	0.88^**^ (0.82, 0.95)	0.86^***^ (0.80, 0.92)	0.74^***^ (0.68, 0.81)	<0.001
Animal-based DDS	1.00	0.93 (0.87, 1.00)	0.94 (0.87, 1.00)	0.92 (0.84, 1.00)	<0.038
Plant-based DDS	1.00	0.91^**^ (0.85, 0.97)	0.76^***^ (0.70, 0.82)	0.64^***^ (0.58, 0.71)	<0.001
**65** ≤ **Age** ≤ **79**					
Total DDS	1.00	0.94 (0.82, 1.06)	0.89^*^ (0.80, 1.00)	0.78^***^ (0.67, 0.90)	<0.020
Animal-based DDS	1.00	0.84^**^ (0.75, 0.95)	0.94 (0.83, 1.05)	0.99 (0.86, 1.14)	<0.037
Plant-based DDS	1.00	0.95 (0.85, 1.06)	0.77^***^ (0.67, 0.87)	0.66^***^ (0.57, 0.78)	<0.017
**80** ≤ **Age** ≤ **99**					
Total DDS	1.00	0.84^***^ (0.78, 0.91)	0.85^***^ (0.80, 0.91)	0.70^***^ (0.64, 0.77)	<0.001
Animal-based DDS	1.00	0.86^***^ (0.80, 0.93)	0.88^***^ (0.82, 0.95)	0.83^***^ (0.76, 0.91)	<0.001
Plant-based DDS	1.00	0.93^*^ (0.87, 0.99)	0.80^***^ (0.74, 0.86)	0.64^***^ (0.58, 0.71)	<0.002
**Age** ≥**100**					
Total DDS	1.00	0.89 (0.77, 1.02)	0.86^*^ (0.76, 0.97)	0.78^**^ (0.67, 0.91)	<0.023
Animal-based DDS	1.00	0.97 (0.85, 1.11)	0.93 (0.81, 1.05)	0.97 (0.83, 1.14)	<0.038
Plant-based DDS	1.00	0.85^**^ (0.76, 0.96)	0.79^***^ (0.69, 0.90)	0.66^***^ (0.55, 0.79)	<0.001

The results were based on Model 4 controlling for gender, age, household income, education level, marital status, pre-retirement occupation, physical activities, smoking, and drinking; DDS, dietary diversity score. ^*^p < 0.05, ^**^p < 0.01, ^***^p < 0.001.

### 3.4 Sensitivity analyses

As shown in Table 4, the same results were seen when Model 4 was used in a sensitivity analysis. After removing people with eye conditions that affect vision like glaucoma, macular degeneration, diabetic retinopathy, or eye injuries (*n* = 7,995), the associations between DDS (the total diet, the animal-based diet, and the plant-based diet) and possible cataracts were similar to the findings in Model 4 of Table 2.

**Table 4:** Sensitivity analysis of DDS with possible cataract.

**Variables**	**Quartiles of DDS**				**P trend**
	**Q1**	**Q2**	**Q3**	**Q4**	
Total DDS	1.00	0.85^***^ (0.80, 0.91)	0.86^***^ (0.82, 0.91)	0.74^***^ (0.68, 0.80)	<0.001
Animal-based DDS	1.00	0.87^***^ (0.82, 0.93)	0.90^***^ (0.85, 0.95)	0.90^**^ (0.84, 0.97)	<0.001
Plant-based DDS	1.00	0.93^**^ (0.88, 0.98)	0.78^***^ (0.73, 0.83)	0.65^***^ (0.60, 0.71)	<0.001

The results were based on Model 4 by removing participants who had other eye conditions that affect vision, including glaucoma, macular degeneration, diabetic retinopathy, and eye injury; Model 4: Adjusted for gender, age, household income, education level, marital status, pre-retirement occupation, physical activities, smoking, and drinking; DDS, dietary diversity score. ^***^, ^**^, and ^*^ represent significance levels of 1%, 5%, and 10%, respectively.

## 4 Discussion

In this study, we investigated the association between dietary diversity and the prevalence of possible cataracts among the Chinese elderly population. We also explored the different patterns of dietary diversity in relation to possible cataracts. It was found that a higher DDS was associated with lower odds of possible cataract, but animal-based diet faced heightened cataract odds which marking an alarming departure from initial trends. Based on subgroup analysis, the animal-based diet showed a significant relationship between DDS and possible cataracts for men, while this association was not significant for women.

Our findings are in line with previous studies that have suggested a link between diet and cataract risk. Several epidemiological studies have reported protective effects of certain dietary components on cataract development (46, 47). However, single nutrients or foods may not reflect the overall quality and adequacy of a diet and may ignore the potential interactions and synergies among different dietary components (48, 49). We discovered associations between heightened dietary diversity and reduced cataract likelihood, even adjusting for confounders. This suggests that savoring an array of foods could be the recipe for robust lens protection. A potential explanation is that dietary diversity may increase antioxidants and nutrients that protect the lens from oxidative damage and inflammation. Oxidative stress has been proved that it can induce cataracts by warping lens protein architecture and function (50). Thus, antioxidants can scavenge free radicals and repair oxidative damage to the lens (17). In addition, inflammatory pathways and cytokines disrupting lens clarity can also catalyze cataracts (51). Therefore, anti-inflammatory nutrients may modulate inflammatory responses and attenuate lens injury (52). The impacts may stem from constituents like phytochemicals and antioxidants which mitigate oxidative stress and inflammation linked to cataract genesis. Abundant bioactive compounds in diverse diets can directly neutralize free radicals, boost endogenous antioxidant systems, and beneficially modulate signaling cascades driving ocular damage. Through attenuating these insults via numerous mechanisms, the synergistic actions of varied nutrition may preserve crystalline lenses. In summary, myriad constituents within diverse foods could synergistically attenuate molecular injuries underlying vision loss.

To capture nutrient interplays and synergies, we examined the different patterns of dietary diversity in relation to possible cataracts. DDS was classified into three categories: total diet, animal-based diet, plant-based diet. We found that a total diet and a plant-based diet can help reduce the risk of cataracts, but an animal-based diet increases the likelihood of cataracts. The development trend of DDS in animal-based diets is exactly opposite to the development trend of the two DDS mentioned above. This suggests that different types of dietary diversity may have different effects on the risk of cataracts. Previous studies have shown that an animal-based diet may increase the risk of cataracts by inducing inflammation or glycation (19, 28–31, 35). Animal-based foods are rich in saturated fat, cholesterol, heme iron, and advanced glycation end products (AGEs), which can increase oxidative stress or inflammation in the lens (17, 50). It is known that AGEs can accumulate in the lens and cause cross-linking or modification of lens proteins, leading to lens opacity (53, 54). Therefore, consuming a high amount of animal-based foods may not be beneficial for preventing cataract development. On the other hand, the plant-based diet may help reduce the risk of cataracts by providing antioxidants or anti-inflammatory nutrients (18, 19, 31). Their phytochemicals, vitamins C and E, carotenoids, flavonoids, polyphenols, and other plant nutrients can mitigate inflammatory responses and oxidative harm (48, 49). Additionally, plant fibers, omega-3s, and other nutrients modulate inflammation and attenuate lens injury (18, 27, 48). Thus, DDS analysis uncovers how food synergies and interactions influence cataract outcomes. Outcomes indicate dietary diversity helps safeguard aging vision, underscoring public health implications. Initiatives which increase affordable produce access may curb projected vision impairment among seniors. Animal foods distinctly lacked pronounced cataract protection seen with fruits/vegetables, although global meat intake rises. Thus diverse traditional cuisines centered on varied plants optimally support ocular health. Integrating these cultural patterns into lifestyle guidelines and interventions could promote elderly vision. In summary, promoting nutritional variety in aging populations carries significant implications for preventing vision loss via clinical recommendations and policy-level action.

A striking finding was the sex divergence in DDS-cataract links. Whereas higher animal-based DDS correlated with heightened cataract risks in men, no such significant association emerged for women (55). One study noted gender discrepancies in cataract prevalence, implicating hormones, genes, lifestyles, and, crucially, diets (17). Among these factors, diet may play an important role in modulating the gender differences in cataract risk. For example, males tend to consume more animal-based foods, while females tend to consume more plant-based foods (56). Males also tend to consume more alcohol and tobacco, while females tend to consume more tea and coffee (57). Such dietary variations could impact antioxidant and nutrient intake relevant to lens health. Moreover, dietary diversity may interact with gender-specific hormones, metabolism, and other physiological factors to influence cataract risk (58). Depending on the levels of oxidative stress markers and inflammatory cytokines involved in lens damage, males and females' oxidative stress and inflammation may be affected differently by dietary diversity (59).

According to the results of this study, it is important to promote a diversified diet to prevent the development of cataract in the Chinese elder group. The plant-based diet that provides antioxidants or anti-inflammatory nutrients is the key to reducing the odds of cataract. The results also point out the significant differences in the association of various diet patterns with possible cataract between men and women. While some studies claim that the animal-based diet causes lens damage, the effect of the animal-based diet on women remains questionable. Although the effect of the animal-based diet on possible cataract of women was not marked in this study, it is likely due to the relatively lower consumption of animal-based foods by women. The interaction between the animal-based diet and the odds of cataract in females requires further research.

The present study has some weaknesses. The cataracts may not match clinical diagnosis since they were identified by self-reported visual function assessment. Thus, we use “possible cataracts” to reflect the subjective nature of this screening approach. As our study lacks long-term, sustained follow-up surveys, we are unable to determine a causal relationship between nutritional diversity and cataract likelihood. Nonetheless, we argue that the discovered results demonstrate robustness, evidenced by multivariate analysis across various demographic groups. Additionally, the voluntary participation design carries risks of selection bias if systematic differences exist between surveyed groups and the broader elderly population that may impact nutrition and cataract development. However, our diverse, cross-regional sample mitigates potential sampling bias. While our study exclusively focuses on elderly Chinese, constraining generalization presently, we advocate for analogous multi-year studies in other ethnic groups to definitively assess nutritional impacts on vision-impairing lens changes. Furthermore, interpreting the divergence in cataract associations between animal and plant diversity requires prudent qualification, given inherent analytic challenges in nutrition epidemiology. Specifically, our food group-based diversity scores may fail to capture complexities within expansive food categories. We encourage future studies to pay closer attention to the intrinsic differences between diverse nutritional sources.

The present study also has multiple strengths. First, a substantial sample of elderly individuals from China was used, which was obtained through a nationwide survey with a commendable response rate. This enhances the generalizability and representativeness of our findings. Second, we used a straightforward and unbiased measure of dietary diversity by assessing the frequency of consumption of ten food groups that match the dietary standards established for Chinese populations. This approach helps us reduce the potential inaccuracies in measurements and the influence of recall bias commonly observed in self-reported dietary consumption data. Third, we adjusted for several potential confounding variables that could affect the relationship between DDS and the risk of developing cataracts. However, this study cannot conclude causality due to the cross-sectional study design and other residual and unmeasured confounding.

Further robust trials are required to confirm the suggested causal links, including cluster studies assigning seniors to interventions targeting total, animal, or vegetable dietary diversity, evaluating resultant ophthalmic changes. Additionally, quantitative intake diaries and repeated clinical eye checks in long-running elderly cohorts could correlate nutrition with lens function over time. Utilizing these prospective methodologies is vital for validating the diet-cataract connections identified here. In summary, randomized controlled trials and prospective cohorts tracking nutritional diversity and quantified vision changes longitudinally are essential next phases.

## 5 Conclusion

In summary, higher DDS was associated with reduced risk of cataract among elderly people in China. This implies that promoting a diversified diet may provide an economical and effective intervention for preventing cataract in the elder group. However, the causal relationship between dietary diversity and cataract risk requires further research.

## Data availability statement

The datasets presented in this study can be found in online repositories. The names of the repository/repositories and accession number(s) can be found below: publicly available datasets were analyzed in this study. This data can be found here: https://opendata.pku.edu.cn/dataverse/CHADS.

## Ethics statement

The studies involving human participants were reviewed and approved by the Ethics Committee of Peking University, Peking University, Beijing, China (IRB00001052-13074). The patients/participants provided their written informed consent to participate in this study. The studies were conducted in accordance with the local legislation and institutional requirements. The participants provided their written informed consent to participate in this study. Written informed consent was obtained from the individual(s) for the publication of any potentially identifiable images or data included in this article.

## Author contributions

HZ: Conceptualization, Data curation, Funding acquisition, Methodology, Writing – review & editing. JZha: Formal analysis, Visualization, Writing – original draft, Validation. JZho: Formal analysis, Visualization, Writing – original draft, Validation. YM: Supervision, Validation, Writing – review & editing.
